# A theory-driven qualitative study exploring issues relating to adherence to topical glaucoma medications

**DOI:** 10.2147/PPA.S174922

**Published:** 2019-05-17

**Authors:** Stephanie McDonald, Eamonn Ferguson, Martin S Hagger, Alexander J E Foss, Anthony J King

**Affiliations:** 1 School of Psychology, University of Nottingham, Nottingham, UK, stephanie.mcdonald@nottingham.ac.uk; 2 School of Psychology, Curtin University, Perth, WA, Australia; 3 Department of Ophthalmology, Nottingham University Hospital, Nottingham, UK

**Keywords:** glaucoma, adherence, patient experience, qualitative research, Common-Sense Model of Self-Regulation

## Abstract

**Purpose:**

Investigating patients’ perceptions of their illness can provide important insights into the experience and management of the illness and associated treatment, and enhance understanding of variations in adherence to prescribed medication. The Common-Sense Model of Self-Regulation (CSM) provides a theoretical framework for the study of illness cognitions, health behavior, and adherence to health recommendations. The aim of this study was to use the CSM to investigate the experience of glaucoma and its treatment from the patients’ perspective, and to apply these insights to classify and clarify issues related to nonadherence with treatment.

**Patients and methods:**

A qualitative investigation using semi-structured interviews took place in two outpatient glaucoma clinics. Thirty-three patients with primary open-angle glaucoma using hypotensive eye drops participated in the study. Deductive content analysis was used to analyze the interview data.

**Results:**

Issues relating to nonadherence with hypotensive eye drops and patients’ experience with their glaucoma and treatment were identified. Treatment schedule and patient factors were classified as common barriers to adherence. Further themes include experienced symptoms of glaucoma, illness coherence, and the emotional and practical consequences of the illness.

**Conclusion:**

Findings provide important insights into the emotional and practical outcomes of glaucoma for patients, perceived symptoms of the illness, and insights into patient memory and cognition. These findings provide supporting evidence for the importance of conducting theoretically driven qualitative investigations of patients’ experience with glaucoma and their treatment, and provide suggestions on key issues that need to be addressed in future multidimensional interventions aimed at improving adherence and patient quality of life.

## Introduction

Glaucoma is the second most common cause of registered blindness in the UK^1^ and a major cause of blindness worldwide, with an estimate of 79.6 million people to be affected by glaucoma by 2020.^2^ Glaucoma is a pressure-related optic neuropathy; currently the only treatable risk factor for glaucoma is increased intraocular pressure (IOP).^3^,^4^ In the majority of patients IOP can be lowered to prevent or slow further vision loss with the use of hypotensive eye drops.^5^,^6^ While the efficacy of antiglaucoma medications is established, a significant proportion of patients prescribed these drops do not adhere to their treatment regimen.^7^ Nonadherence to treatment affects the individual, in terms of disease progression and visual disability, as well as society with valuable resources required to pay for unused medications and additional interventions to achieve disease control, along with support for those with preventable glaucoma-related disability.^8^,^9^

The majority of studies in the literature investigating the antecedent factors relating to adherence to glaucoma treatment have employed a quantitative methodological approach,^10^–^12^ with fewer studies, by comparison, adopting a qualitative approach.^13^–^16^ A qualitative approach allows for an in-depth analysis of patient experience and provides a method for identifying concerns and needs that are not always evident from quantitative methods.^17^,^18^ While previous qualitative research has identified factors relating to nonadherence with glaucoma treatment, one of the critiques in the literature is the lack of sufficient theory-driven investigations to inform patient-centered interventions, which aim to enhance adherence.^19^–^21^ Health behavior theories provide evidence-based insights of key determinants of behavior.^22^ Research has shown that developing a theoretical understanding of factors that influence behavior, and the underlying mechanisms of this process, is essential in the design and implementation of effective interventions of behavior change to target those factors.^22^–^24^ The use of a qualitative method of inquiry offers researchers the means to explore how prespecified theory-based influences of behavior are experienced and construed by patients.

The Common-Sense Model of Self-Regulation (CSM) provides a theoretical framework for the study of individuals’ illness representations (or illness perceptions) and how these may guide the adoption of coping strategies to manage their illness.^25^ According to the CSM, individuals form a cognitive representation of a health threat, which consists of the following five dimensions: identity, consequences, cause, timeline, and cure/control.^26^ Identity reflects perceived symptoms and illness label. Consequences relate to beliefs regarding the impact of the illness on a person’s life. Timeline is the perceived duration of the illness. Cause is defined as individuals’ beliefs relating to causal factors of the illness. The cure/control dimension reflects beliefs about whether the illness can be cured or controlled.^26^ This has subsequently been divided into personal control, to reflect beliefs relating to the controllability of the illness by the individual’s actions, and treatment control associated with beliefs regarding the effectiveness of treatment in controlling or curing the illness.^27^ A sixth dimension, illness coherence representing individuals’ understanding of their illness, was also included thereafter in measures of illness perceptions (eg, the Revised Illness Perception Questionnaire).^27^ Individuals also form emotional representations, in a parallel process, which reflect affective reactions to the health threat.^28^ Cognitive and emotional representations guide the adoption of coping strategies to manage the illness and the emotional response to the illness, and these in turn influence illness and emotional outcomes.^29^ Problem-focused coping strategies, such as treatment adherence, are likely to lead to adaptive outcomes such as effective management of the illness, better functioning, and quality of life.^29^ Individuals’ appraisal of the efficacy of such coping mechanisms can lead to a modification of illness representations.^28^

The CSM is regarded as a dominant theoretical model in explaining health outcomes and providing insights into how individuals manage a chronic illness in their everyday life;^25^,^28^ and studies have shown associations between patients’ illness perceptions and adherence to treatment.^30^ However, only a limited number of studies have investigated illness perceptions in relation to medication adherence in patients with glaucoma,^19^,^31^,^32^ and these have utilized a quantitative methodological approach. The present study extends previous research and adds to the available literature by closely adhering to the CSM as a theoretical framework to guide the investigation. Using the CSM provides the basis to explore how illness perceptions, which have previously been shown to affect adherence, are construed by patients with glaucoma through their narrative of their experience with glaucoma and prescribed treatment. Adopting the CSM enables the identification of factors that are potentially modifiable, such as illness representations or perceived barriers, which could be targeted to enhance adherence to treatment.^33^ The application of the CSM to these issues will contribute to formative research that will inform the development of multidimensional interventions to improve medication adherence in patients with glaucoma. Interventions designed to change patients’ perceptions of their illness have been associated with positive health outcomes and improved adherence to treatment.^34^,^35^ The emphasis on theory in the present study means that we are able to provide recommendations of interventions that are grounded in theory. The primary aim of our study was to explore patients’ perceptions and experience of their glaucoma and treatment regimen, and how these cognitions may relate to variations in adherence to treatment. We adopted innovative methods to provide a comprehensive, detailed description of factors contributing to medication adherence in patients with glaucoma. We present thematic maps of patients’ experience with their illness and treatment based on dimensions of the CSM and taxonomies identified in the literature in regard to barriers to adherence.^13^–^16^ A further aim was to provide suggestions of appropriate interventions, based on our findings, and to identify issues, which may require further investigation. As this is a qualitative investigation, we do not report specific hypotheses, but expect dimensions from the CSM to feature in the data, offering key insights of patients’ perceptions and experience with their illness.

## Materials and methods

### Ethics

Ethical approval for the present investigation was granted by the NHS Nottingham Research Ethics Committee 2 (Reference: 10/H0408/38). Written informed consent was obtained from all patients taking part in the study prior to data collection. Participants were informed that they could withdraw from the study at any point during the interview. All patients providing consent participated fully in the interview and their data were included in the analysis.

### Interview schedule

The interview schedule was based on the Brief Illness Perception Questionnaire,^36^ a validated measure of patients’ illness perceptions across a number of illnesses. The questions were designed to capture patients’ views and experiences relating to the identity of the illness, perceived consequences, cure/control, illness coherence, and emotional representations. These were supplemented with additional questions concerning patients’ self-reported adherence to treatment and issues with treatment regimen, which were informed by prior literature. Five general questions were included in the schedule (Table 1). For each question, additional probing questions were prepared prior to the study to clarify patients’ responses and to provide a detailed account of their experience with their illness. All interviews were conducted by the same researcher, and these lasted ~20 minutes.

### Analysis protocol

**Figure 1:**
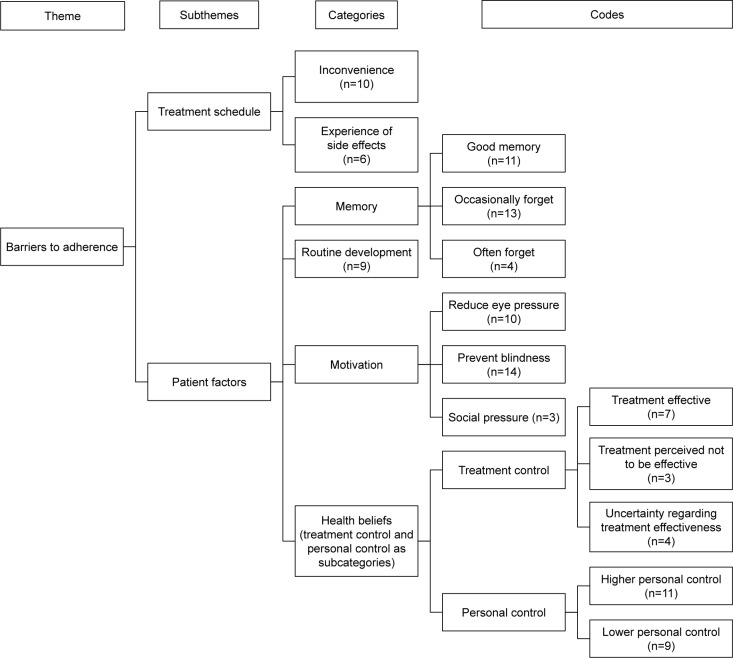
Thematic map featuring “barriers to adherence”. **Note:** n represents the number of interview transcripts containing the specified codes.

**Figure 2:**
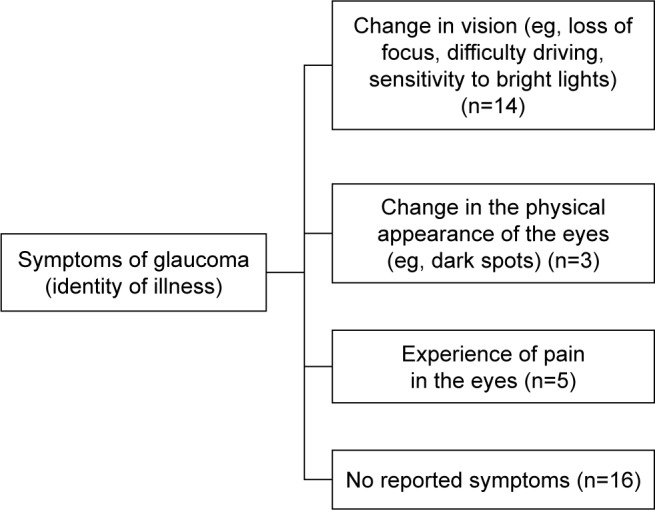
Thematic map of “symptoms of glaucoma”. **Notes:** The theme “Symptoms of glaucoma” consists of four categories: “change in vision”, “change in the physical appearance of the eyes”, “experience of pain in the eyes”, and “no reported symptoms”. n represents the number of interview transcripts containing the category-relevant codes.

**Figure 3:**
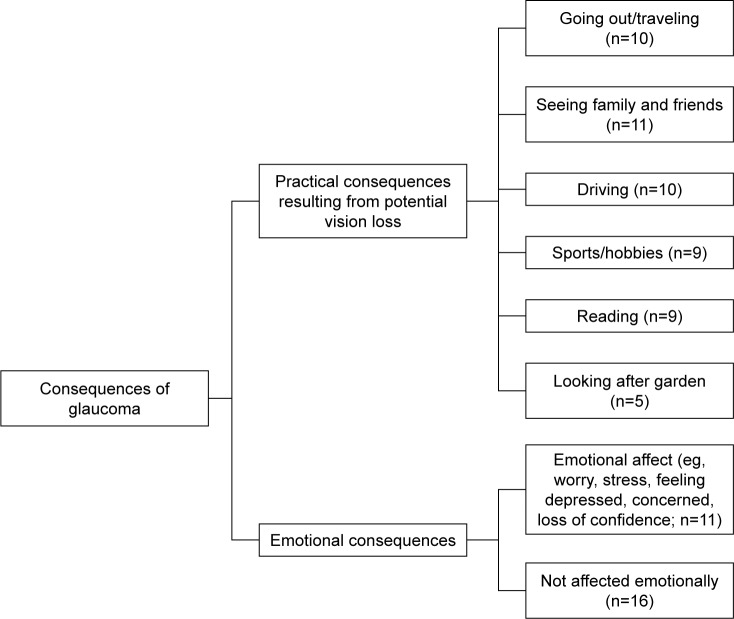
Thematic map featuring “consequences of glaucoma”. **Notes:** The theme “consequences of glaucoma” was described by two subthemes, “practical consequences resulting from potential vision loss” and “emotional consequences”, and their associated categories, as identified in patients’ interviews. n represents the number of interview transcripts containing the category-relevant codes.

Thirty interviews were audio-recorded and transcribed verbatim; for the remaining three interviews, notes were taken of participants’ narratives during the interview, and these were used in the analysis. Interview transcripts were imported into the NVivo8 software,^37^ a program for organizing data in order to conduct qualitative content analysis.^38^ Deductive content analysis was adopted, which involves the analysis of patients’ transcribed accounts driven by a theoretical construct (ie, the CSM) and previous findings in the literature.^13^–^16^ This comprised three phases: preparation, organizing, and reporting of the analysis and findings.^38^ These are described below and the resulting content categories and derived themes are presented in the form of thematic maps (Figures 1–3).

### Preparation phase

The preparation phase involved the reading of transcripts before starting the analysis.

### Organizing phase

This phase involved classifying text from the interview transcripts into content categories in order to develop themes relating to our research question. This took the form of a top-down process. The CSM dimensions and findings from previous literature investigating patient experience and adherence with glaucoma treatment were used to guide the development of categories. Patients’ responses were then reviewed to identify key words of short phrases, which were coded on the basis of the developed categories. Manifest coding was used, meaning that patients’ exact responses were coded. Codes were then assigned to each relevant category. For example, in regard to the “identity of the illness”, patients’ narratives revealed the experience of symptoms that were attributed to glaucoma, such as blurred vision and sensitivity to bright lights (codes). These codes that reflected a change in patients’ vision were grouped, together with similar others, into the category of “change in vision”. The frequency of occurrence of the identified categories was counted once per transcript, rather than how many times a specific code was seen in a specific interview transcript.

Categories were reviewed and refined to ensure that there was no overlap and to assess whether two or more categories could be integrated when frequency of occurrence in the transcripts was low. This process also enabled the researchers to identify underlying psychological concepts linking several categories together in order to derive themes of patients’ narratives. Themes were developed by grouping categories together based on their content and meaning in relation to the research question. Depending on the nature of the categories, some of the identified themes had associated subthemes linked to them, and these were organized in a hierarchy.

The resulting themes and their associated subthemes and categories were verified by an independent rater to ensure reliability.^39^ Results showed substantial agreement between the researcher and the independent rater (κ=0.74, *P*<0.001).

### Reporting phase

Following the identification of categories, subthemes, and themes, thematic maps were developed to illustrate the content and nature of the relationship between them.

## Results

Thirty-three patients (20 males, 13 females) with an average age of 70.7 years (SD =12.32; range: 31–90 years) took part in the study. Inclusion criteria included having visual acuity >6/12 and a diagnosis of ocular hypertension or primary open-angle glaucoma requiring treatment with hypotensive eye drops. Patients reported taking antiglaucoma medications for an average of 7 years (range: 1 month to 20 years). Nine patients reported having undergone glaucoma-related surgery.

Four themes were developed, which explained patients’ beliefs and experience with their illness and treatment: 1) barriers to adherence, 2) symptoms of glaucoma, 3) consequences of glaucoma, and 4) illness coherence. These are presented below, together with selected quotations from the interview transcripts.

### Barriers to adherence

Two subthemes were developed under the theme of barriers to adherence, which illustrate common obstacles that patients face, which may prevent them from taking their eye drops as prescribed. These were treatment schedule and patient factors (Figure 1). The rationale for this was that issues with treatment and issues to do with patients’ lifestyle, motivation, and health beliefs (under patient factors) were factors associated with barriers to adherence, and this is supported by previous literature.^13^,^16^

#### Treatment schedule

Treatment schedule refers to difficulties experienced by patients, which may prevent them from taking their eye drops as prescribed. The majority of patients reported not experiencing any issues with their treatment schedule, while a number of patients found the eye drops inconvenient to use (n=10). This was related to design issues of the bottles, the number and frequency of drop application, as well as having other competing tasks that clashed with the time of day that patients had to take their medication. One patient reported “They are not easy to put in, they have me an eye drop stopper but it floods your eye going in, there’s more than is intended” (male, 70). Other patients mentioned “… it’s inconvenient when you have to do it four times a day and if you have to go out for the day and you don’t take your drops with you …” (female, 75) and “[some eye drops] are supposed to be kept in the fridge which is deeply inconvenient if you are out somewhere…” (male, 69). Patients also reported the experience of medication side effects, such as stinging of the eyes or blurred vision.

#### Patient factors

This refers to issues related to patients’ beliefs regarding their illness and treatment, and their ability to adhere with treatment. Four categories were developed that were directly related to patient factors: memory, based on self-reported prior adherence, routine/organizational skills in taking the eye drops, motivation, and health beliefs.

In regard to memory issues, one third of the patients reported having good memory, in that they reported never having missed doses since they were prescribed the medication. However, the majority of patients (n=17) reported that they would forget to take their eye drops, and this ranged from occasionally forgetting to often forgetting to take medication as prescribed. A number of patients mentioned in their narrative that they would routinely take them as prescribed, while some patients (n=9) specified the development of a system or routine in taking their eye drops, as a device to improve adherence. For example, as one participant stated, “I routinely do it last thing at night like cleaning my teeth, preparing for bed…” (male, 65). Taking drops was linked with food preparation and activities, which acted as reminders to take their eye drops at certain times of the day. As one patient mentioned, other strategies to aid memory included, “… I make columns in my notebook so I make sure I don’t miss them” (female, 76).

A further category of patient factors was motivation in taking the eye drops. Patients reported that they took their eye drops to reduce eye pressures. Patients also reported that they believed that it was important to take their eyes drops in order to prevent loss of vision. As one patient mentioned, “I feel I’ve got no choice, if I don’t take them I feel I’ll go blind…” (male, 70). Social pressure, in terms of doctors checking eye pressures and family members reminding patients to take their eye drops, were reported to motivate patients to take their eye drops.

Health beliefs, such as personal control and treatment control, were also identified in the interview transcripts. In line with the CSM of self-regulation, treatment control was conceptualized as patients’ perceptions of the efficacy of their treatment in controlling their glaucoma. Patients who stated explicitly in their interview that they felt that their treatment was effective in lowering their eye pressures or preventing further vision deterioration, for example, were seen as having higher treatment control (n=7). For example, one patient reported “… since I started putting drops in my vision to me is perfect … they’ve absolutely done me good that’s all I can say” (male, 83), while another commented “… it’s necessary to bring the pressure down … they found this medication to counteract the pressure and in doing so it’s for my betterment” (male, 65).

Three patients, however, reported losing faith in their eye drop efficacy (reflecting lower treatment control). They felt that their medication failed to lower their eye pressures sufficiently, which, for the majority of these patients, resulted in surgery. One patient reported, “the drops stopped working … that’s why they needed to do the operation … I was curious as to why I need to keep taking them if they are not actually working…” (male, 31). Another patient stated, “I don’t see how they can be effective in my personal experience, if I don’t use the drops for two weeks I don’t notice a significant change in my vision” (female, 51).

Personal control, reflecting patients’ beliefs in whether they can control or manage their illness, in terms of taking medication as prescribed, was observed in the data. One third of patients reported that they felt confident that they could administer their eye drops to control their illness, reflecting higher perceived personal control over their illness. Some patients reported that they relied on others to administer their drops or to remind them to take their drops at the right times. As one patient reported, “I’m not very good at putting drops in myself, so my wife puts my drops in usually; I’m a bit cautious with touching my eyes and things like that…” (male, 59).

### Symptoms of glaucoma

This reflects patients’ experience of symptoms attributed to glaucoma during the course of their illness, and as such, the identity they ascribed to their illness (Figure 2). A general consensus (n=14) in the interviews was that patients experienced change in vision as a result of glaucoma such as loss of focus, difficulty in reading distant material, and sensitivity to bright lights. A small number of patients (n=3) reported that they experienced changes in the physical appearance of their eyes as well as pain in the eyes (n=5), as a result of glaucoma. Approximately half of the patients reported having no glaucoma-like symptoms. Some felt that the absence of symptoms indicative of the illness may be a factor contributing to nonadherence: “… people are not taking their drops because there are no symptoms, they don’t know that the pressures are up…” (female, 65).

### Consequences of glaucoma

Patients were asked to describe the potential impact of glaucoma on their lives. Perceived consequences of glaucoma were identified as a theme, as shown in Figure 3. This was described in terms of practical outcomes of the illness, reflecting both current and future anticipated consequences, and emotional consequences of living with glaucoma.

In regard to practical consequences, inability to travel as a result of vision deterioration (n=10) was a prominent identified code in patient transcripts: “… suddenly you can’t drive and take buses … independence …” (male, 70). Other patient reports included not being able to see family and friends (n=11). Patients also reported that they would miss pursuing their hobbies such as reading (n=9), watching TV (n=8), watching or participating in sports (n=9), and looking after their garden (n=5).

In regard to the emotional consequences of the illness, one third of patients mentioned that they were concerned about their glaucoma. Worry, anxiety, and loss of confidence were commonly reported outcomes. Patients reported “I’m on the edge of worrying, sometimes I can’t even sleep at night…” (male, 79), “It’s depressing because you can’t see” (female, 78) and that “It would be traumatic to lose your eye sight…” (male, 48). Others reported that they were constantly aware of the problem (“… you always have glaucoma at the back of your mind…”, female, 66), and some experienced disappointment and loss of confidence in carrying out daily activities. For example, one patient mentioned, “I feel disappointment that I have to use the drops…” (male, 73), while another commented that “… I don’t really have the confidence now to cope with situations on the road” (female, 64).

### Illness coherence

Illness coherence reflects clarity and level of certainty that patients have with their illness, and an overall understanding of their illness and treatment. This was developed from responses to questions associated with what patients have learnt from their experience of glaucoma, how their life changed as a result of their diagnosis, and how they felt about using their eye drops.

Results showed that a number of patients expressed knowledge or an awareness of the nature of their glaucoma, such as for example, increased eye pressures, visual field loss, which was associated with their illness, as well as knowledge of the function of prescribed treatment. The illness coherence theme was developed by coding transcripts with explicit detail on the nature of their illness and the function of medication. Thus, for patients who were seen as having higher illness coherence, there was explicit evidence in their narrative of the aforementioned points. Responses included “It’s a build-up of pressure at the back of the eyes and damages nerves which leads to blindness” (male, 64). Similarly, another patient reported, “What I’ve learned in all fairness, the pressures can be reduced with the drops” (male, 49).

The majority of patients spoke about their illness in general terms, or reported that they were unsure as to what the illness entailed, or the function of their eye drops, thus showing through their narrative, lower illness coherence. For example, one patient said “I’m only using them, because I’m told to use them … to reduce the strain off my eyes, I’m not sure what they meant there is a strain in my eyes” (male, 65). A similar issue was raised by another patient: “I was very concerned because I don’t know enough about it” (male, 59). These findings suggest that exploring patients’ understanding of their condition is important to determine possible misconceptions in relation to the illness or treatment.

## Discussion

The present investigation used a dominant theoretical framework, the CSM, to develop an assessment of patients’ experience with glaucoma that goes beyond a commonly used evaluation, and includes a broader range of factors that may be related to treatment adherence and quality of life. The CSM was also used to organize the findings of this study. This study adds to the literature by providing important insights on the emotional outcomes of the illness, patients’ perceptions of glaucoma-related symptoms and adverse events relating to the medication, insights into illness coherence, and memory as a key contributor to self-reported nonadherence. This is the first study, to the authors’ knowledge, which has attempted to link the barriers to adherence with these other factors in a coherent and structured way.

Adopting the CSM to guide the current analysis assists in informing the development of optimally effective interventions to modify nonadherent behavior. In regard to interventions to address issues of adherence in patients with glaucoma, a Cochrane review^40^ concluded that “due to inadequate methodological quality and heterogeneity of study design we are unable to advocate any particular interventions at this time” (p. 2). Further patient-centered interventions are needed based on patients’ perceptions of their illness and treatment regimen. Findings from the present study indicate that interventions to improve adherence should take into consideration a comprehensive view of patients’ experience and illness perceptions. This study identified potential multifactorial components that may influence adherence, one or more of these may affect a patient, and the strategies developed must envelop these different components into a comprehensive package of adherence support.

### Emotional consequences of the illness

A key finding in the present investigation was mood and depression as emotional consequences of the illness. When patients were asked to report the consequences of their illness, findings revealed that a number of patients were emotionally affected by the illness. Some patients reported feeling depressed, stressed, and worried about their illness. Thus, mood and depression emerged as key emotional outcomes in the present investigation. This presents a key finding, as emotional consequences, such as depression, have previously been shown to have an effect on nonadherence to health recommendations. A meta-analysis (N=47) investigating the relationship between depression and adherence to treatment found that patients with depression were more likely to be nonadherent.^41^ The relationship between depression and anxiety, and nonadherence to treatment has also been observed in patients with glaucoma.^42^,^43^ In fact, studies have shown that “even nonclinical levels of depressive symptoms can be associated with non-adherence” (p. 2402).^44^ This suggests that depression must be recognized as a key risk factor for poor health and nonadherence to health recommendations. The present investigation served to identify these emotional outcomes in patients with glaucoma. Interventions aimed at improving adherence and quality of life in patients with glaucoma should include an element to address emotional distress.

### Practical consequences of the illness

In addition to emotional consequences, patients also expressed their concerns with the practical consequences of their illness on their lifestyle and quality of life as a result of potential vision loss due to glaucoma; this relates to the consequences dimensions of the CSM. Such concerns included inability to drive, to see family and friends, and pursue their hobbies, among others. It is likely that the importance of different aspects of vision loss is different between patients. A concern however emerging from the interview transcripts was loss of independence because of visual failure. This is in the context of an aging population when other aspects of aging such as loss of hearing and limited mobility may also be affecting patients’ ability to maintain their independence. A study investigating views of glaucoma patients on their treatment^45^ identified that loss of driving license (rather than blindness) was the most important aspect of treatment success for patients. Using the information derived from patients’ experiences, concerns and lifestyle will allow relevant tailored patient-specific interventions to be generated with health messages that incorporate the most important concerns. Such concerns, derived from patients’ self-reported experiences (eg, inability to drive, read, watch TV, visit family), should be focused on patients’ health concerns and used in health messages that would act to motivate adherence.^46^ As such, these would serve as powerful motivators to minimize medication nonadherent behavior.

### Memory

Barriers to adherence included a range of factors relating to treatment schedule and patient-centered issues, which were seen as inhibiting factors for adhering to prescribed eye drops. The identified barriers to adherence confirm previous findings.^13^–^16^ Perceived barriers are likely part of the control dimension of the CSM and, therefore, interventions to overcome them are likely to result in better adherence. Identifying such issues early on in treatment could enable clinicians to offer better education to patients and solutions to such issues if necessary. Educating patients to deal with commonly reported obstacles and enhancing health beliefs associated with the importance of medication can improve patient adherence.

In regard to patient factors, the majority of patients reported occasionally forgetting to take eye drops, especially when faced with competing activities or traveling away from home. For some patients, such issues were overcome by developing a routine linked with everyday activities such as taking medication in combination with meals or activities that served as cues. This insight may provide a mechanism to improve adherence in other patients if useful habit-forming associations can be identified and their role in improving adherence developed. Perhaps the assessment of patients with glaucoma should include enquiry about their routine daily activity to identify if their drop dosing schedule could be incorporated into an already established routine and stimulated by a “trigger activity” such as brushing teeth and this could be enhanced by ensuring that drops are located beside their toothbrush, for example. This could form part of implementation intention and action-planning strategies aimed at enhancing adherence.^47^,^48^

### Identity of illness

In regard to the perceived identity of illness, commonly reported symptoms were changes in vision and physical appearance of eyes as well as pain in the eye area. It is unlikely that these symptoms are caused by glaucoma per se as most patients, unless their IOP is significantly elevated, are unaware of their elevated IOP. It is more likely that patients are confusing some of the adverse effects of medication, which may feel like “pressure in the eye” with a true sensation of raised pressure. Thus, experiencing these side effects may act as a demotivating factor for taking their medication, in order to avoid the effects. In such cases, awareness of such adverse events would initiate a change in medications producing no or less side effects, thus enhancing the chance of adherence. Informing the patient on the nature of symptoms that they may experience as a result of their glaucoma, as well as identifying adverse events, which may be side effects from the medication, could help to improve adherence to treatment and increase patient quality of life. Furthermore, given the absence of symptoms in the majority of patients with glaucoma, other techniques may be needed in this nonsymptomatic group to enhance adherence, such as education regarding the risks of nonadherence and the benefits of preserving good vision.

### Limitations

A limitation of the present investigation relates to the small sample size used. This was dictated by achievement of theme saturation, which is consistent with previous studies using qualitative methods. However, the sample selected provided an adequate representation of patient experience of glaucoma and issues related to treatment.

## Conclusion

The present study used a dominant theoretical model in the study of treatment adherence, the CSM of Self-Regulation, to explore patient-specific parameters, as identified in the constructs that patients presented in their interviews, relating to how patients represent their illness and how these processes may guide decisions relating to treatment. We have also used this framework to organize the findings in the form of thematic maps. Findings from this investigation guided by the CSM can inform the design of future patient-centered interventions to overcome patient-specific issues related to poor adherence based on patients’ illness perceptions. Further, these findings provide supporting evidence of the need to conduct theoretically driven qualitative investigations of patients’ experience with their illness and treatment, and using the identified themes and subthemes to provide recommendations for the development of future patient-specific interventions. This study has identified barriers to adherence, perceptions of glaucoma-related symptoms and adverse events of medications, perceived consequences of the illness, and provided insights on what issues need to be addressed in a single multidimensional intervention aimed at improving adherence to glaucoma treatment and patient quality of life.

## Figures and Tables

**Table 1:** Interview schedule

Questions	Prompts
1. From your experience, what do you think the symptoms of glaucoma are?	• Have you experienced any of these symptoms?
2. What have you learnt from this experience?	• Prophylaxis • Do you feel it is important to take your medication as prescribed? Why/why not? • Any issues with the treatment schedule?
3. How do you feel about using your eye drops?	• If patients mentioned development of a routine to help them take their eye drops, they were asked to specify the nature of the routine • Self-administration of eye drops or help from others? • How often do you miss your drops? Occasionally/often/never • Reasons for using/not using eye drops, if a patient suggests that they are adherent/nonadherent
4. What are the consequences of your glaucoma on your daily life, if any?	• Prevents you from carrying out certain tasks that would otherwise be possible? • Does it affect you emotionally? • Negative consequences of further vision deterioration/vision loss?
5. How has your life changed after being diagnosed with glaucoma?	• For better (awareness) or worse (obstacles to face in daily life)?

**Note:** Interview schedule with five predetermined questions and prompts associated with each question.
